# Suicidal Ideation of Men with Alcohol Use Disorder in South Korea: A Structural Equation Modeling Analysis

**DOI:** 10.3390/ijerph18073353

**Published:** 2021-03-24

**Authors:** Hyon Joo Hong, Sung Hee Shin

**Affiliations:** 1 National Center for Mental Health, Seoul 04933, Korea; hhong0325@gmail.com; 2 College of Nursing Science, Kyung Hee University, Seoul 02447, Korea

**Keywords:** alcohol use disorder, suicidal ideation, impulsivity, life stress, structural equation modeling, South Korea

## Abstract

South Korea’s suicide rate has been ranked second among OECD countries, and the rate of suicidal ideation is the highest among men with alcohol use disorder. To test a hypothetical model of men with alcohol use disorder based on O’Connor’s integrated motivational-volitional model, a study was conducted at a psychiatric outpatient clinic, a community addiction management center, and an Alcoholics Anonymous meeting in South Korea, comprising 203 men with alcohol use disorder. Data were collected using the Scale for Suicide Ideation, Barratt Impulsiveness Scale-11, Childhood Trauma Questionnaire-Short Form, Survey of Recent Life Experiences-Short Form, Defeat Scale and Entrapment Scale. The final model was a good fit to the data (*χ*^2^/df = 1.51, comparative fit index = 0.97, normed fit index = 0.92, incremental fit index = 0.97, Tucker–Lewis index = 0.96, and root mean square error of approximation = 0.05). The structural model explained 34.0% of the variance in suicidal ideation; and it validated that impulsivity, stress, defeat, and entrapment were the key factors affecting suicidal ideation. To prevent suicide among men with alcohol use disorder, it is necessary to develop a suicide prevention program that includes men’s feelings of defeat and entrapment.

## 1. Introduction

South Korea’s suicide rate has been ranked second among Organization for Economic Cooperation and Development countries [1]. Suicide prevention, which is an important policy goal of South Korea’s national mental health promotion program, is a sociopolitical issue that should be treated seriously [2]. In particular, the suicide rate in South Korea is 28.7 per 100,000, which is more than double that of other OECD [1] countries (i.e., average of 12.1 per 100,000). Most (60–90%) of those who die from suicide have mental disorders, and the rate of lifetime suicidal ideation in South Korea is 15.4%, whereas the rate of lifetime suicide attempts is 2.4% [3]. Among individuals with mental disorders, the rates of suicidal ideation and attempts are the highest among men with alcohol use disorder.

Individuals with alcohol use disorder have reduced abilities to control emotions when they drink because rational judgment is often impaired after alcohol consumption; therefore, acute or chronic alcohol abuse acts as a triggering event for suicide [4]. Further, the rate of suicide is 2.6 times higher among South Korean men than women [5], and the rate of lifetime suicidal ideation in men with alcohol use disorder (28.3%) is more than double that of women with alcohol use disorder (12.3%) [3].

Previous studies in South Korea about suicidal behavior in those with alcohol use disorder include characteristics of those who have attempted suicide [6,7] and validated factors related to suicide [8,9,10,11]. However, no study has validated the various paths leading to suicide. Moreover, exploration of the progress toward suicide motivation has been limited.

According to previous studies, individuals with alcohol use disorder experience family issues, financial instability, and social issues; and they also have difficulty in forming and maintaining close social relationships with other individuals, which leads to social, psychological, and emotional isolation [6]. These emotions are influenced by certain predispositions, such as mental disorders and alcohol use disorder [12]. Individuals with vulnerability factors including aggression, impulsivity, and perfectionism [13] are more heavily influenced by environment and life events, such as childhood trauma, current life events, interpersonal stress, and social isolation [14]. Social defeat refers to the perception of isolation and reduced self-worth in society [15], and entrapment occurs when the affected individual wishes to overcome the defeat but realizes that they cannot do so. Subsequently, to overcome the pain and problems caused by alcohol addiction, individuals attempt suicide. This may lead to a vicious cycle of alcohol addiction and attempted suicide [6]. Therefore, to identify factors that influence suicide in individuals with alcohol use disorder, an integrated approach should consider cognitive factors—such as defeat and entrapment—as well as background factors and potential triggering events such as vulnerability factors, environmental factors, and life events.

Suicide occurs due to a complex interaction of various factors, thus various approaches have been taken to explore the factors affecting suicide—such as biological, psychological, sociological, etc. [16]. The components of O’Connor’s [17] integrated motivational-volitional model were influenced by other theories. This model aimed to supplement existing suicide theories that had been explained from a narrow perspective. To this end, the theory established the progression from distal risks in the past to recent proximal psychological risks and then to suicidal behavior into a theoretical framework, while also maintaining previous theoretical structures [18]. The model thus includes various biological/social factors, such as individual characteristics and environment, and cognitive factors, such as defeat and entrapment, and it provides an appropriate explanation of the complexity of suicide [16]. Through this, the path toward suicidal ideation that reflects the characteristics of alcohol use disorder can be confirmed from an integrated perspective.

In order to develop an intervention to prevent suicidal ideation, since repetitive suicide ideation may result in suicide attempts and death by suicide [19], this study aimed to establish and validate a structural equation model consisting of factors that influence suicidal ideation in men with alcohol use disorder, based on O’Connor’s [17] integrated motivational-volitional model. Through this, the study aimed to provide theoretical evidence for the development of effective nursing strategies to prevent suicide among those with alcohol use disorder.

## 2. Materials and Methods

### 2.1. Theoretical Framework and Hypothetical Model

**Figure 1:**
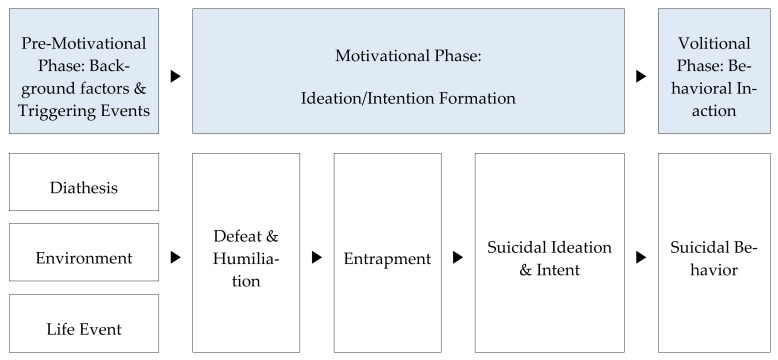
Theoretical framework.

Based on previous research findings, this study established a hypothetical model that includes factors previously found to be related to suicidal ideation in those with alcohol use disorder as well as defeat and entrapment, which are included in O’Connor’s [17] integrated motivational-volitional model (Figure 1). The integrated motivational-volitional model combines three theories—diathesis-stress hypothesis, arrested flight model, theory of planned behavior—and approaches suicide from an integrated perspective that includes an overall view of the complex interaction between biological, psychological, environmental, and cultural factors. Through this, the model established a theoretical map showing how important psychological risk factors are converted to suicidal behavior through a series of phases [18] and explained suicidal behavior in the following three phases: pre-motivational, motivational, and volitional phases.

O’Connor’s [17] integrated motivational-volitional model was influenced by other theories in organizing its components. The motivational-volitional model takes an integrated approach and includes various biological, social, and cognitive factors; therefore, it provides an appropriate explanation of the complexity of suicide and is readily applicable [16]. Among those with alcohol use disorder, the factors that lead individuals to choose suicide are not limited to their current situations. Instead, individuals experience defeat in society, such as in family, work, and interpersonal relationships, owing to current alcohol use stemming from negative past experiences. Such defeat does not resolve alcohol use disorder and eventually leads to suicide [7]. Moreover, individuals cannot solve social problems arising from repeated drinking and instead avoid problems by relying on drinking. Therefore, they think of suicide as an escape from reality [20]. The integrated motivational-volitional model can be applied as a model that reflects past and current background factors and suicidal ideation caused by cognitive factors in individuals with alcohol use disorder.

O’Connor’s [17] integrated motivational-volitional model includes suicide attempts in the volitional phase. However, the present study validated the structural equation model results for suicidal ideation in the motivational phase. Therefore, based on previous research, impulsivity [21,22], childhood trauma [23,24], and stress [25,26] were set as latent variables, background factors, and triggering events. We constructed a hypothetical model and verified its suitability to explain the suicidal ideation among men with alcohol use disorder. Moreover, we investigated the direct and indirect effects of factors influencing suicidal ideation. 

### 2.2. Structural Equation Modeling (SEM)

This study aimed to test the suicidal ideation process in men with alcohol use disorder based on the theory of the O’Connor’s [17] integrated motivational-volitional model. Therefore, structural equation modeling (SEM) was the most appropriate statistical equation method for this study.

SEM is a statistical technique for verifying the causal relationship between variables in the form of a combination of confirmatory factor analysis and path analysis [27]. In SEM, latent variables are measured indirectly through observed variables that are directly measured. An observed variable, used as an indirect measure of a construct, is an indicator, and the statistical realization of a construct based on analyzing scores from its indicators is a factor. The capability to analyze observed or latent variables in SEM, as either causes or outcomes, permits great flexibility in the types of hypotheses that can be tested [28].

### 2.3. Study Design and Participants

This was a cross-sectional study. Participants were adult men aged 19–64 years who were diagnosed with alcohol use disorder by a psychiatrist and currently attending community addiction management centers, psychiatric outpatient clinics, or Alcoholics Anonymous. The minimum sample size for maximum likelihood—which is often used in structural equation modeling—is usually between 100 and 150, and the recommended sample size is approximately 200 [29]. The sample size was 203 in this study, thus satisfying the minimum recommended sample size.

### 2.4. Data Collection Procedure

Data were collected between 5 February and 16 March 2018. The researcher explained the study purpose to hospital directors or department heads and sought cooperation, and questionnaires were subsequently distributed and collected. Prior to data collection, the researcher explained the purpose and methods of this study to potential participants and also orally explained that participants could withdraw from the study whenever they wished without any disadvantages. Then, participants were asked to provide written consent on a form that included the aspects that were explained orally prior to completing the surveys. For ethical protection of study participants, this study was approved by the institutional review board at the Hospitaller Order of St. John of God institution (no. 2017-9).

### 2.5. Measurements

#### 2.5.1. Suicidal Ideation

To measure suicidal ideation, we used the Scale for Suicide Ideation, which was developed by Beck, Kovacs, and Weissman [30] and translated into Korean and converted into a self-reported questionnaire format by Shin, Park, Oh, and Kim [31]. It consists of 16 items across 3 subdomains: active suicidal desire, passive suicidal desire, and suicidal ideation. Each item is rated on a scale of 0 to 2, with higher scores indicating higher suicidal ideation. Overall Cronbach’s α was 0.87 [31]; however, reliability for each subdomain was not provided. In this study, Cronbach’s αs were 0.91 for the entire tool, and 0.86 for active suicidal desire, 0.61 for passive suicidal desire, and 0.76 for suicidal ideation.

#### 2.5.2. Impulsivity

For impulsivity, the 11th edition of the Barratt Impulsiveness Scale [32], translated into Korean by Lee [33], was used. It consists of 23 items across 3 subdomains: motor impulsiveness, cognitive impulsiveness, and non-planning impulsiveness. Each item is rated on a 4-point Likert scale ranging from 1 (strongly disagree) to 4 (strongly agree). Negative items were scored in reverse. Higher scores indicate higher impulsivity. According to Lee [33], Cronbach’s αs were 0.81 for the tool, 0.70 for motor impulsiveness, 0.73 for cognitive impulsiveness, and 0.50 for non-planning impulsiveness. In this study, Cronbach’s αs were 0.90, 0.83, 0.69, and 0.78, respectively.

#### 2.5.3. Childhood Trauma

Childhood trauma was measured using the Childhood Trauma Questionnaire-Short Form, which was first shortened by Bernstein et al. [34] to 28 questions and then translated into Korean and shortened to 20 items by Kim and Kim [35]. It consists of four subdomains: emotional abuse, physical abuse, emotional neglect, and physical neglect. Each item is rated on a 4-point Likert scale from 1 (never) to 4 (often), and negative items are reverse scored. Higher total scores indicate a higher level of childhood trauma. Cronbach’s α ranged from 0.91 to 0.95 at the time of development and was 0.89 in Kim and Kim [35], who reported separate Cronbach’s αs of 0.86 and 0.85 for abuse and neglect, respectively. In this study, Cronbach’s αs were 0.89 for the tool, 0.82 for emotional abuse, 0.81 for physical abuse, 0.91 for emotional neglect, and 0.53 for physical neglect.

#### 2.5.4. Stress

Stress was measured based on the Survey of Recent Life Experiences-Short Form, developed by Kohn and Macdonald [36]. The current researcher translated the tool into Korean according to processes for translating instruments into other languages, as suggested by Hilton and Skrutkowski [37]. The scale was developed for adults to measure perceived stress in daily life. It consists of 41 items across 6 subdomains: social and cultural difficulties, work, time pressure, finances, social acceptability, and social victimization. Each item is rated on a 4-point Likert scale ranging from 1 (never) to 4 (very often), and higher scores indicate higher levels of stress. At the time of development, Cronbach’s αs were 0.90 for the tool, 0.78 for social and cultural difficulties, 0.82 for work, 0.81 for time pressure, 0.76 for finances, 0.68 for social acceptability, and 0.76 for social victimization. In this study, Cronbach’s αs were 0.94, 0.82, 0.89, 0.79, 0.76, 0.83, and 0.82, respectively.

#### 2.5.5. Defeat

Defeat was measured using 12 items from the Defeat Scale, developed by Gilbert and Allan [38] and revised and translated into Korean by Lee et al. [39]. Each item is scored on a 5-point Likert scale from 1 (strongly disagree) to 5 (strongly agree). Higher total scores indicate higher levels of defeat. At the time of development, Cronbach’s α was 0.93; in Lee et al. [39], it was 0.93; and, in this study, it was 0.97.

#### 2.5.6. Entrapment

Entrapment was measured using 16 items from the Entrapment Scale, developed by Gilbert and Allan [38] and translated into Korean by Lee and Cho [40]. It comprises two subdomains: external entrapment, which explains current situations or relationships; and internal entrapment, which explains current emotions or thoughts. Each item is scored on a 5-point Likert scale from 1 (strongly disagree) to 5 (strongly agree). Higher total scores indicate higher levels of entrapment. At the time of development, Cronbach’s α was not presented for the tool. Instead, it was presented for each subdomain: 0.89 for external entrapment and 0.86 for internal entrapment. Similarly, Lee and Cho [40] only presented Cronbach’s αs for each subdomain: 0.92 for external entrapment and 0.89 for internal entrapment. In this study, Cronbach’s α were 0.95 for the tool, 0.93 for external entrapment, and 0.91 for internal entrapment. 

### 2.6. Statistical Analysis

The collected data were analyzed using SPSS Windows version 23.0 and AMOS version 21.0 (IBM Corporation, Armonk, NY, USA). General characteristics of participants and characteristics of the study variables were presented as descriptive statistics, such as mean, standard deviation, frequency, and percentage. Tool reliability was confirmed through Cronbach’s α, and the normality of the study variables was confirmed through normalized skewness and kurtosis. A confirmatory factor analysis was conducted to confirm the validity of the factors, and convergent, discriminant, and nomological validity was confirmed. To evaluate model fit, chi-squared tests (*χ*^2^), the root mean square residual, comparative fit index, normed fit index, incremental fit index, Tucker–Lewis index, and root mean square error of approximation were used. Bootstrapping was used to test the significance of the indirect and total effects of the study model.

## 3. Results

### 3.1. Demographic and Disorder-Related Characteristics

Participants’ mean age was 51.4 years, and most had a high school degree and were married. More participants were employed than unemployed, and many earned less than 1,000,000 KRW per month. Most (71.4%) started drinking before they were aged 19 years. The drinking level at the time of participation was measured using the Korean version of the Alcohol Use Disorders Identification Test. As suggested by Oh et al. [41], the cutoff was 15 points: 54.7% of participants scored <15 and 45.3% scored ≥15. Nearly one-third (31.5%) had a family history of alcohol use disorder, and 12.3% had a family history of suicide. Nearly one-third (31.5%) had previously attempted suicide, and 58.1% were receiving outpatient care (Table 1).

### 3.2. Descriptive Statistics and Test of Normality of the Observed Variables

Participants’ mean scores for the observed variables were as follows: 2.35 for impulsivity (range 1–4), 1.77 for childhood trauma (range 1–4), 1.73 for stress (range 1–4), 2.60 for defeat (range 1–5), 2.55 for entrapment (range 1–5), and 0.47 for suicidal ideation (range 0–2). In the evaluation of the normality of the observed variables, normality was assumed when the absolute value for skewness was less than 3 and that for kurtosis was less than 10. Here, the kurtosis score should be interpreted after adding three [29]. In this study, the absolute values for skewness ranged between 0.05 and 0.93; thus, they were smaller than three. The values obtained by adding three to the absolute values for kurtosis ranged between 3.13 and 4.20 (i.e., <10). In other words, these values indicated that the observed variables were normally distributed (Table 2).

### 3.3. Testing Model Fit of the Hypothetical Model

To confirm whether the collected data appropriately explained the hypothetical model of the study in a structural equation model, a two-step approach was employed. In the first stage, the relationship between the observed variables and each latent variable was confirmed through a confirmatory factor analysis. Subsequently, in the second stage, the overall fit of the hypothetical model and the significance of the path were evaluated [42].

#### 3.3.1. Confirmatory Factor Analysis of Observed Variable

Through a confirmatory factor analysis, variables with a factor loading ≥ 0.50 and significance (t) of ≥1.96 were selected, while other measurement items that did not satisfy these criteria were removed. Through this, 13 out of 16 suicidal ideation items, 19 out of 23 impulsivity items, and 18 out of 20 childhood trauma items were selected. The time pressure was removed from the six stress subdomains, and 25 out of the 41 items were selected. For defeat, 11 out of 12 items were selected, and 15 out of 16 entrapment items were selected. In summary, of the 128 items, 101 were selected for analysis.

The defeat factor used in this study was a single factor. To obtain convergent and discriminant validity when using the defeat factor in the confirmatory factor analysis in the same format as other factors, the observed variables for defeat were parceled into two item parcels through item-to-construct balancing [43]. In other words, the variables were ordered in the magnitude of factor loading and were assigned, in an alternating manner, into the two item parcels in groups of two. These were then calculated as summed variables and used in the confirmatory factor analysis model. Factor loading, average variance extracted, and composite construct reliability were used to ensure the validity of factors. Factors with a factor loading ≥0.5, an average variance extracted ≥0.5, and a composite construct reliability ≥0.7 were assumed to have convergent validity [27]. The variables in this study had factor loadings that ranged 0.6–0.95, average variance extracted that ranged 0.62–0.92, and composite construct reliability that ranged 0.86–0.97, thus satisfying the criteria.

#### 3.3.2. Test of the Hypothetical Model

The model was assumed to have a good fit when *χ*^2^/df was <3, root mean square residual was ≤0.05, comparative fit index was ≥0.90, normed fit index was ≥0.90, incremental fit index was ≥0.90, Tucker–Lewis index was ≥0.90, and root mean square error of approximation was ≤0.05 [27]. The analysis showed that the model fit was good (*χ*^2^/df = 1.51, root mean square residual = 0.03, comparative fit index = 0.97, normed fit index = 0.92, incremental fit index = 0.97, Tucker–Lewis index = 0.96, and root mean square error of approximation = 0.05).

### 3.4. Testing of Hypothetical Model and Analysis of Effects

**Figure 2:**
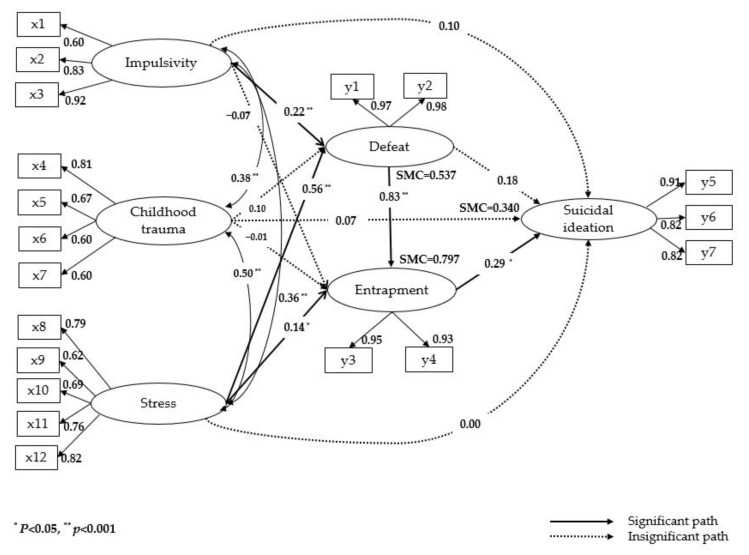
Path diagram for the modified model. x1 = motor impulsiveness; x2 = cognitive impulsiveness; x3 = non-planning impulsiveness; x4 = emotional abuse; x5 = physical abuse; x6 = emotional neglect; x7 = physical neglect; x8 = social and culture difficulties; x9 = work; x10 = finances; x11 = social acceptability; x12 = social victimization; y1 = total defeat 1; y2 = total defeat 2; y3 = external entrapment; y4 = internal entrapment; y5 = active suicidal desire; y6 = passive suicidal desire; y7 = suicide plan.

The analysis of the model supported 5 out of 12 hypotheses. The path diagram for the modified model is presented in Figure 2. Impulsivity (β = 0.22, *p* = 0.001) and stress (β = 0.56, *p* = 0.001) had direct effects on defeat, and impulsivity and stress explained 53.7% of the variance in defeat. Defeat (β = 0.41, *p* = 0.001) and stress (β = 0.14, *p* = 0.014) had direct effects on entrapment, and stress (β = 0.46, *p* = 0.001) and impulsivity (β = 0.18, *p* = 0.003) had indirect effects. These factors explained 79.7% of the variance in entrapment through direct and indirect effects. Entrapment (β = 0.29, *p* = 0.041) had a direct effect on suicidal ideation, and stress (β = 0.28, *p* = 0.006) and defeat (β = 0.42, *p* = 0.002) had significant total effects. Moreover, impulsivity (β = 0.07, *p* = 0.046) and stress (β = 0.28, *p* = 0.001) had indirect effects, indicating that these completely mediated suicidal ideation through defeat and entrapment. The direct and indirect effects of these factors explained 34.0% of the variance in suicidal ideation (Table 3).

## 4. Discussion

The purpose of this study was to construct and test a hypothetical model to explain suicidal ideation of men with alcohol use disorder based on O’Connor’s [17] integrated motivational-volitional model. This study found that impulsivity, childhood trauma, stress, defeat, and entrapment, which are factors that affect suicidal ideation in men with alcohol use disorder, explained 34.0% of the variance in suicidal ideation, with a good model fit.

The results indicate that entrapment has a direct effect on suicidal ideation in men with alcohol use disorder, and defeat indirectly affected suicidal ideation through partial mediation by entrapment. As such, defeat is a strong factor that influences entrapment and increases suicidal ideation in men with alcohol use disorder. This finding is in line with previous reports that defeat and entrapment in participants with a history of trauma influence suicidal ideation [44]; that patients with more serious positive symptoms of schizophrenia experience more defeat and entrapment, which increases suicidal ideation [45]; and that reduced problem-solving and lack of social support increase defeat and entrapment, thus increasing suicidal ideation and behavior [46].

Defeat and entrapment are core concepts of psycho-social cognitive processes that are used to explain suicidal ideation and behavior in the Williams [47] Cry of Pain model, and suicide is regarded as the only way to escape from the unbearable pain caused by defeat and entrapment. Here, suicidal behavior is assumed to be a motivation to escape from itself owing to entrapment [48]. Moreover, the arrested flight model explains that individuals experience defeat as a response to humiliation and rejection. The individual then experiences entrapment as they cannot find an alternative solution for the problem and choose suicide to solve this issue [48]. As such, suicide is considered the only way to escape from the unbearable pain caused by defeat and entrapment. Therefore, defeat, entrapment, and suicide are closely related and are continuous processes. An individual perceives defeat and entrapment owing to their circumstances, and defeat and entrapment affect suicidal ideation and behavior; therefore, defeat, entrapment, and suicide are closely related. In particular, individuals with alcohol use disorder experience social issues, and they also have difficulty forming and maintaining intimate social relationships with friends, colleagues, and other individuals, which leads to social and psychological emotional isolation. Moreover, to overcome the pain and problems caused by alcohol addiction, individuals with alcohol use disorder attempt suicide using alcohol. Following this, individuals live in a cycle of alcohol addiction and attempted suicide [6]. It is believed that the defeat and entrapment of alcohol use disorder are key cognitive factors in suicidal ideation. As such, providing interventions for defeat and entrapment in alcohol use disorder is an important factor in preventing suicide.

Stress is another factor that affects entrapment and increases suicidal ideation in men with alcohol use disorder. This coincides with previous findings that individuals experience entrapment in stressful situations [49,50]. Life events causing stress often occur before suicide attempts, and suicidal ideation from these events leads to suicide attempts [51]. In other words, life events that cause stress act as triggering events, and individuals have suicidal ideation when they cannot escape from these situations.

In particular, those with alcohol use disorder are exposed to various stresses as it progresses to addiction, experiencing family conflict, economic instability, and social problems. Moreover, persons with alcohol use disorder experience various forms of stress in the community, such as at home and work, and experience entrapment in which they wish to escape from stressful situations but are unable to. It is believed that this leads to suicidal ideation as a means of resolving this situation. Moreover, alcohol use disorder has an impulsive diathesis, and individuals may attempt to cope with stressful situations through heavy drinking. Future studies should confirm the various types and levels of stress in individuals with alcohol use disorder and repeat research on the effect of stress on entrapment.

Another factor that affects defeat and entrapment in men with alcohol use disorder and increases suicidal ideation is impulsivity. This finding is in line with previous studies reporting that impulsivity affects defeat and entrapment [52,53]. According to the integrated motivational-volitional model, vulnerability factors in the pre-motivational phase play a role in times of extreme stress and create a biosocial background that can lead to suicidal ideation and suicidal behaviors [17]. Vulnerability is a characteristic similar to personality and includes factors such as despair, impulsivity, perfectionism, optimism, and resilience [13]. In alcohol use disorder, impulsivity is known to be a typical personality predisposition [54]. As such, individuals with alcohol use disorder often display impulsivity and are thus likely to attempt suicide out of impulse rather than with a clear plan [55]. Moreover, those who attempt suicide out of impulse often choose highly fatal methods, such as poisoning or jumping off a high structure, rather than hanging [56]. Therefore, suicidal ideation should be more carefully treated in individuals with impulsive characteristics, especially among those with alcohol use disorder. According to a recent study by O’Connor [57], alcohol factors can play an important role in transforming self-harm thoughts into self-harm behavior. In this context, preventing suicidal ideation among those with alcohol use disorder, who are unable to control drinking, may help reduce suicide risk.

Previous studies reported that defeat and entrapment influenced suicidal ideation in the general public [58], individuals with trauma experience [44], individuals with self-harm behavior [59], and patients with schizophrenia spectrum [45]. This study confirmed that defeat and entrapment are important factors that increase suicidal ideation in men with alcohol use disorder, which had not been researched in previous studies.

Nonetheless, this study had some limitations. First, the SEM path generally presents ‘causality’, but the data were collected cross-sectionally and therefore, no firm conclusions can be made about causal inferences. Second, since this study examined individuals with alcohol use disorder from specific local and institutional communities in South Korea, generalization of the current findings to all persons with alcohol use disorder requires caution. Third, there are also limitations in applying the study’s findings to women with alcohol use disorder or those in other age groups, such as children, adolescents, and older adults with alcohol use disorder. Therefore, based on the present findings, future comparative studies should apply the factors that influence suicidal ideation in men with alcohol use disorder to women and other age groups. In addition, it is necessary to develop and test the effects of nursing intervention programs for suicidal ideation in men with alcohol use disorder, including the factors confirmed in this study.

## 5. Conclusions

To confirm the factors that influence suicidal ideation in men with alcohol use disorder, this study developed a hypothetical model based on O’Connor’s [17] integrated motivational-volitional model and validated the model fit with the collected data. The model was a clear, appropriate model that can explain suicidal ideation in South Korean men with alcohol use disorder. Entrapment had a direct effect on suicidal ideation in men with alcohol use disorder, and impulsivity and stress had indirect effects on suicidal ideation through the mediation of defeat and entrapment. The findings indicate that individuals with higher impulsivity and stress experience more defeat and entrapment, which increases suicidal ideation. Therefore, to prevent suicidal ideation—which is on a continuous spectrum with suicidal behavior—in men with alcohol use disorder, a specialized suicide prevention program that includes defeat and entrapment as core concepts and specialized nursing strategies that are appropriate for men with alcohol use disorder should be developed.

## Figures and Tables

**Table 1:** General Characteristics of Participants (*N* = 203).

Variables	Categories	*N*	%	M ± SD
Age (year)	20–39	29	14.3	51.40 ± 10.30
	40–49	49	24.1	
	50–59	68	33.5	
	60–64	57	28.1	
Education level	≤Elementary school	26	12.8	
	Middle school	29	14.3	
	High school	90	44.3	
	≥College	58	28.6	
Marital status	Divorce	41	20.2	
	Not divorce	162	79.8	
Job	Employed	115	56.7	
	Not employed	88	43.3	
Monthly income (10,000 won)	<100	121	59.6	
	≥100	82	40.4	
Early onset of drinking (year)	≤19	145	71.4	18.11 ± 4.19
	20–29	52	25.6	
	≥30	6	3.0	
Drinking level ^†^	<15	111	54.7	13.91 ± 11.71
	≥15	92	45.3	
Family history of AUD	yes	64	31.5	
	no	139	68.5	
Family history of suicide	yes	25	12.3	
	no	178	87.7	
Suicide attempt experience	yes	64	31.5	
	no	139	68.5	
Outpatient clinic	yes	118	58.1	
	no	85	41.9	

^†^ Korea version of Alcohol Use Disorder Identification Test (AUDIT-K).

**Table 2:** Descriptive Statistics and Confirmatory Factor Analysis of Measurement Model (*N* = 203).

Variable	Range	M ± SD	Skewness	Kurtosis	AVE	CCR
Impulsivity	1–4	2.35 ± 0.52	−0.05	0.27	0.81	0.93
Childhood trauma	1–4	1.77 ± 0.59	0.93	0.71	0.62	0.86
Stress	1–4	1.73 ± 0.52	0.79	0.20	0.71	0.92
Defeat	1–5	2.60 ± 1.18	0.12	−1.20	0.87	0.97
Entrapment	1–5	2.55 ± 1.00	−0.11	−0.99	0.87	0.93
Suicidal ideation	0–2	0.47 ± 0.48	0.92	−0.13	0.92	0.97

AVE = average variance extracted; CCR = composite construct reliability.

**Table 3:** Standardized Direct, Indirect, and Total Effects of the Modified Model (*N* = 203).

Exogenous Variables	Endogenous Variables	Direct Effect (*p*)	Indirect Effect (*p*)	Total Effect (*p*)	SMC
Impulsivity	Defeat	0.22 (0.001)		0.22 (0.001)	0.537
Childhood trauma		0.10 (0.083)		0.10 (0.083)	
Stress		0.56 (0.001)		0.56 (0.001)	
Impulsivity	Entrapment	−0.07 (0.068)	0.18 (0.003)	0.11 (0.175)	0.797
Childhood trauma		−0.01 (0.413)	0.09 (0.097)	0.07 (0.361)	
Stress		0.14 (0.014)	0.46 (0.001)	0.60 (0.002)	
Defeat		0.83 (0.001)		0.83 (0.001)	
Impulsivity	Suicidal ideation	0.10 (0.102)	0.07 (0.046)	0.18 (0.073)	0.340
Childhood trauma		0.07 (0.226)	0.04 (0.136)	0.11 (0.304)	
Stress		0.00 (0.497)	0.28 (0.001)	0.28 (0.006)	
Defeat		0.18 (0.143)	0.24 (0.052)	0.42 (0.002)	
Entrapment		0.29 (0.041)		0.29 (0.041)	

SMC = squared multiple correlation.
